# Types of Community Support Services and Self-Efficacy for Continuous Community Living among Individuals with Disabilities and Caregivers

**DOI:** 10.3390/ijerph191912976

**Published:** 2022-10-10

**Authors:** Wai Chan, Yuan Cao, Erin Yiqing Lu, Wai Ming Cheung, Hector Wing Hong Tsang

**Affiliations:** 1 Department of Rehabilitation Sciences, The Hong Kong Polytechnic University, Hung Hom, Hong Kong; 2 Mental Health Research Centre, The Hong Kong Polytechnic University, Hung Hom, Hong Kong; 3 Faculty of Education, University of Hong Kong, Pokfulam, Hong Kong

**Keywords:** individuals with disabilities, caregivers, self-efficacy for continuous community living, center-based services, financial subsidy

## Abstract

This study explored the impacts of five types of community support services (i.e., center-based care, home-based care, respite care, caregiver assistance, and financial subsidies) on self-efficacy for continuous community living among individuals with disabilities and caregivers. Design: Cross-sectional. Method: The sample consisted of a group of individuals with disabilities (*n* = 948) and a group of caregivers (*n* = 522). A mixed ANOVA was applied to explore the differences in the perceived importance of improvements to community support services between the groups. Logistic regression analyses were conducted to examine the perceived importance of improvements to types of community support services for self-efficacy for continuous community living. Results: Caregivers perceived higher levels of importance for improvements to community support services than individuals with disabilities. Both groups reported that financial subsidies were the most important area for improvement. The greater importance of improvements to financial subsidies reported by caregivers predicted greater odds for self-efficacy for continuous community living. The greater importance of improvements to center-based services reported by individuals with disabilities predicted greater odds for self-efficacy for continuous community living. Conclusions: The findings suggested that financial subsidies for caregivers and center-based services for individuals with disabilities could improve self-efficacy for continuous community living.

## 1. Introduction

Community integration for individuals with disabilities has been advocated for decades. Starting in the 1950s, the deinstitutionalization movement in the United States and Europe proposed that inpatients with disabilities had the right to live in the community [1]. In 2006, the United Nations officially announced the right to community living among people with disabilities, and the right to get access to community support services [2,3]. As of the 2010s, only 45% of the world’s nations have implemented deinstitutionalization [4] and continuous community living. In this day and age, we need to ask how and why this transition to community living is still “a work in progress” [5] or even “ill-regulated” [6].

The pros and cons of the deinstitutionalization of individuals with disabilities have been previously documented. Deinstitutionalized individuals with disabilities can enjoy a better quality of care and improved adaptive behaviors, but they may experience poorer physical/mental health and interpersonal problems [7,8,9]. Studies investigating the extent to which individuals with disabilities who are living in the community are able to integrate and how their well-being can be improved during community living have shown inconsistent findings [7]. Moreover, the factors contributing to community living are still inconclusive.

Community living can yield various outcomes for different groups of stakeholders, such as individuals with disabilities. One possible determining factor is the availability of support services in the community. Service gaps are often barriers to community living, for example, some community services are inadequate or the expectations for programs are not clear [10]. Nearly 50% of caregivers for children with intellectual disabilities failed to use services that were suggested by professionals, whereas more than 30% of caregivers stated that the suggested services did not meet their needs [11]. Some service users were not aware that certain services could be accessed in the community or that formal financial support was available [12,13]. These gaps in service provision and usage are not uncommon. 

Community services can maximize the independence of individuals with disabilities who are living in the community by providing adequate support to sustain the benefits of community integration. Cheung and Ngan [14] showed that individuals with disabilities in Hong Kong who reported a greater use of social services had a better knowledge of community services, had more desire to learn about the community, and participated in a greater variety of community activities, which could contribute to community integration. However, the gains from community integration may only increase for up to a year after deinstitutionalization and then gradually level off; hence, community services should act as a booster to protect these gains. Community services should be continuously provided or their availability should be increased [8]. 

As well as their importance to individuals with disabilities, community services are also crucial for caregivers in various contexts. The mental health of caregivers has been proposed as the top priority for the provision of support services in Australia [15]. Moreover, the economic well-being and mental health of caregivers has been accounted for by community services and support in the United States [16]. For example, respite care services were able to facilitate stress reduction among caregivers for adult family members with intellectual disabilities in a Chinese speaking population [17]. A study conducted in eastern Europe demonstrated that home-based respite services could provide temporary relief to caregivers, so the provision of services should be increased or at least maintained while care-recipients are living in the community [18]. Conversely, a systematic review reported that a scarcity of social services, such as financial support, could undermine care planning among aging caregivers [19]. 

The present study built on prior research into the impacts of community services on community living among individuals with disabilities and caregivers. The primary aim of the current study was to identify whether and how community support services contributed to self-efficacy for continuous community living among individuals with disabilities and caregivers in Hong Kong. The support services included center-based, home-based, respite care, caregiver assistance, and financial subsidy services. According to census data published in 2021 [20], there were 534,200 people with a disability in Hong Kong. More than 85% were living at home but only 42.7% of them had a caregiver to assist with their daily living, e.g., going out, health care, etc. Some services are available via formal care or non-governmental organizations (NGOs), e.g., transport services by Rehabus [21]. Service use eligibility is often evaluated by professionals. Even though most individuals with a disability were residing in the community, it has been reported that the notion of community living seemed to be imposed in Hong Kong and that their viewpoints on community integration were not well heard [22]. Another study even indicated that some carers in Hong Kong would rather have their care-recipients with intellectual disabilities move in an institution than live at home [23]. We should query how and why carers and people with disabilities come to agree on community living. Additional investigations into the perceptions of stakeholders need to be conducted. 

Moreover, self-efficacy for continuous community living is often regarded to be dynamic throughout life spans. The demand for institutionalized care or residential placements in Hong Kong increases over time, even among those who are already integrated into the community, likely due to declines in the physical health and functioning of the care-recipients or caregivers over time [22,23]. Self-efficacy for community living may vary according to the individual’s limitations, which may be offset by support services in the community. 

Additionally, it would be of substantial practical importance for policymakers to explore how groups of stakeholders evaluate the types of community support services. Groups of stakeholders have their own priorities for resources [24], but the particular types of resources that are favored by given groups of stakeholders have not been explored. Therefore, another aim of this study was to compare the impacts of different types of community service resources on individuals with disabilities and caregivers. The findings could inform policies and processes so that appropriate services can be tailored to the needs of specific stakeholder groups.

More importantly, addressing the relationships between types of community support services and self-efficacy for continuous community living among individuals with disabilities is indispensable to sustainable service allocation and disability care planning. Our shared assumptions suggested that community support services generally encourage community integration, but little empirical evidence is available in the literature. It is logical to hypothesize that some types of community support services may be perceived by different stakeholders to contribute differently to continuous community living [25]. In this study, we aimed to identify which types of community support services, if any, contributed to self-efficacy for continuous community living among a given group of stakeholders and how. The current study also sought to fill knowledge gaps by extending the lines of inquiry to examine community living among individuals with disabilities and caregivers. 

## 2. Methods

### 2.1. Design

The current study was based on a subset of quantitative data from a government consultancy project led by the corresponding author on long-term rehabilitation care in the Hong Kong Special Administrative Region (HKSAR) [26]. The project conducted needs assessments with the aim of developing recommendations and formulating plans to improve the well-being and quality of life of individuals with disabilities and their caregivers through policymaking over the coming decade. 

### 2.2. Participants

The respondents were recruited from more than 300 non-government organizations (NGOs), services units, and self-help associations for people with disabilities (total sample = 1879). Individuals with disabilities who were living in the community and their caregivers, including those who were not members of the same family and could be regarded as two unrelated groups, were eligible for the quantitative part of the parent project. Mental illnesses (20.3%), intellectual disabilities (19.3%), and physical disabilities (19.1%) were reported to be the three main types of disabilities among the subsample of individuals with disabilities for the larger consultancy project [26]. Surveys were distributed in paper form between September and October 2019. Service unit professionals and staff were relied upon for assistance during survey completion whenever necessary.

In the current study, individuals with disabilities (*n* = 948) and caregivers (*n* = 522) who provided fully completed surveys were included in the subsequent analyses. Surveys with missing values were excluded from the analyses, leaving a final sample size of 1470.

### 2.3. Measures

#### 2.3.1. Community Support Resources (Five Dimensions)

The types of support resources were treated as major predictors in the current study. The wide array of services within the local context of Hong Kong were assessed by 12 items, including “to increase the number of District Support Centers (DSCs) for persons with disabilities”, “to increase the quota of Home Care Services”, and “to increase the quota of Day Training Services” (see Appendix A for the full list). Respondents were asked to rate the importance of improvements to each service on a 5-point Likert scale (1 = *not important at all*; 5 = *very important*). A higher value indicated a higher perceived importance of improving that service. 

Based on the service classifications implemented by the Hong Kong SAR, the items were categorized into five dimensions: center-based services (five items), home-based services (one item), respite services (two items), caregiver assistance (two items), and financial subsidies (two items) (see Appendix A). The mean score was computed for each dimension. 

#### 2.3.2. Self-Efficacy for Continuous Community Living (One Dimension)

The parent study outcomes were based on the extent to which respondents were confident in continuing to reside in the community. Based on their opinions and the well-being of the care-recipients with disabilities, the respondents were asked to indicate how long they (i.e., the care-recipient and/or caregiver) could remain living in the community (i.e., self-efficacy for continuous community living) on a 5-point Likert scale (0 = *0 year*; 4 = *more than 10 years*), taking into account the implementation of or improvements to the aforementioned community support services. A higher value indicated a greater inclination to continue to live in the community. To create a binary outcome for the following analyses, item responses indicating 0 years were recoded as 0 (*not self-efficacious for living in the community*) and all other item responses indicating more than 0 years were recoded as 1 (*self-efficacious for living in the community*). 

### 2.4. Covariates

Four covariates (i.e., age, gender, job status, and caregiving support for the respondent) were included in the analyses of the care-recipient and caregiver groups. Age was recoded as an interval variable (from 0 = *younger than 10 years* to 8 = *80 years or above*). Gender (0 = *female*; 1 = *male*), job status (0 = *no current job*; 1 = *has a part/full-time job*), and caregiving support (0 = *no voluntary caregiver*; 1 = *has a voluntary caregiver*) were entered as dichotomous covariates. 

Six additional dichotomous covariates were added in the analysis of the caregiver group to further adjust for their caregiving experiences and the characteristics of the care-recipient(s): years of caregiving (0 = *fewer than* *15 years*; 1 = *15 years or more*), hours of caregiving a day (0 = *12 or fewer hours a day*; 1 = *more than 12 h a day*), the number of care-recipients with disabilities (0 = *only one care-recipient with a disability*; 1 = *has more than one care-recipient with a disability*), the presence of a substitute caregiver if needed (0 = *no*; 1 = *yes*), age of the care-recipient(s) (0 = *younger than 20 years*; 1 = *20 years or above*), and having another care-recipient without a disability (0 = *no*; 1 = *yes*). 

### 2.5. Statistical Analyses

Several statistical analyses were conducted. First, descriptive statistics were calculated to understand the sociodemographic characteristics of the respondents and examine the proportion of respondents who were self-efficacious for living in the community versus those who were not self-efficacious. The ANOVA assumptions were examined using Box’s M test, Mauchly’s W test, and Levene’s test. Data transformation was attempted when assumptions were violated to restore normality; otherwise, appropriate statistical tests were used to maximize robustness as an alternative when data transformation was not effective for the current set of data (Huynh–Feldt epsilon test; [27]). A mixed ANOVA (repeated measures) was conducted to explore differences in the preferences for community service resources between the groups (i.e., the two groups and five types of community service resources), after checking the assumptions. The main effects of group (i.e., between-group) and types of community service resources (i.e., within-group) were also examined. 

Logistic regression modeling techniques were applied to predict self-efficacy for continuous community living by group. The five main types of community support services were entered as predictors. To deal with multicollinearity, the factors of the five community support services were grand mean centered. Respondent sex, age, and employment status and the presence of caregiving support were statistically controlled in all models. The model for the caregiver group was further adjusted by the six other aforementioned covariates. The degree of model fit was assessed by the Hosmer and Lemeshow test [28,29]. Model indices (model χ^2^, Cox and Snell *R*^2^, Nagelkerke *R*^2^, and likelihood ratio index) were used to describe the approximate proportions of the variance that were accounted for by the predictors [30]. In addition, odds ratios (ORs) with confidence intervals that were specific to given predictors were reported to indicate the effects of the factors. 

## 3. Results

The participant characteristics are summarized in Table 1 and Table 2. The respondents were on average older than 40 years. Over 50% of care-recipients were male and about 80% of caregivers were female. More than two thirds of the individuals with disabilities and caregivers were unemployed. Both groups reported the importance of improving all types of community service resources (*M*s > 4.00). Most respondents reported self-efficacy for community living, provided that there was support from community services (i.e., 95% of individuals with disabilities and 95.6% of caregivers), suggesting that sufficient community support services enabled stakeholders to live in the community. 

The second aim of this study was to explore the between- and within-group differences regarding the types of community support services. Given that the study sample included caregivers and individuals with disabilities who could experience long-term distress, their service needs were likely to be very high, which could be reflected in the perceived importance of improvements to services. Thus, the data were inevitably skewed, even after multiple attempts at data transformation. Box’s M test, Mauchly’s W test, and Levene’s test were all statistically significant, indicating that the ANOVA assumptions were hardly met. To minimize biases, the Huynh–Feldt epsilon test was used on the reported data (Leech et al., 2005) instead of data transformation. Despite the unmet assumptions, ANOVA was still considered to be one of the most robust analyses for group differences. 

Based on the mixed ANOVA (repeated measures), caregivers generally reported higher levels of the perceived importance of improvements to services compared to care-recipients (*F*(1, 1464) = 62.07, *p* < 0.001, partial η^2^ = 0.04; grand M_caregivers_ = 4.43, grand M_individuals with disabilities_ = 4.14). There were also differences in the perceived importance of service improvements between the types of support resources (Huynh–Feldt epsilon: *F*(3.60, 1545.35) = 5.98, *p* < 0.001, partial η^2^ = 0.004). Based on the post-hoc Bonferroni corrections, improvements to financial subsidies were perceived to be more important than improvements to other types of support services (M_financial subsidy_ = 4.45 versus M_other services_ ≤ 4.25). The main effects of services were also qualified by interactions between service type and group (Huynh–Feldt epsilon: *F(*3.60, 1545.35) = 10.01, *p* < 0.001, partial η^2^ = 0.01), suggesting that caregivers generally reported higher levels of the perceived importance of service improvements than individuals with disabilities and that the within-group differences between the types of services significantly varied in magnitude by cubic and fourth-order trends (cubic: *F(*1, 1464) = 8.76, *p* < 0.01, partial η^2^ = 0.01; fourth-order: *F*(1, 1464) = 14.65, *p* < 0.001, partial η^2^ = 0.01). These findings demonstrated that the individuals with disabilities and caregivers evaluated the types of community support services differently and that the perceived importance of improvements among each group also varied in magnitude by the type of community support services.

The primary aim of this study was to explore the relationships between community support resources and self-efficacy for continuous community living. According to the bivariate correlation matrices (Table 3 and Table 4), the perceived importance of improvements to center-based services was positively correlated with self-efficacy for continuous community living among individuals with disabilities, whereas the perceived importance of improvements to financial subsidies was positively correlated with self-efficacy for continuous community living among caregivers. In addition, younger caregivers tended to be more self-efficacious for living in the community than their older counterparts (*r* = −0.14, *p* < 0.01).

To compare the relative contributions of the types of community support services, logistic regression was conducted in each group (Table 5). Overall, the logistic regression model explained a modest amount of the variance in the outcomes (individuals with disabilities: model χ^2^ = 12.83, *df* = 9, *ns*; Cox and Snell *R*^2^ = 0.013; Nagelkerke *R*^2^ = 0.041; likelihood ratio index = 0.03; caregivers: model χ^2^ = 21.07, *df* = 15, *p* = 0.134; Cox and Snell *R*^2^ = 0.040; Nagelkerke *R*^2^ = 0.130; likelihood ratio index = 0.11). The model fits were typical (individuals with disabilities: Hosmer and Lemeshow test = 3.17, *df* = 8, *ns*; caregivers: Hosmer and Lemeshow test = 10.03, *df* = 8, *ns*).

Odds ratios were estimated to explore the unique contributions of the various types of community support services. For individuals with disabilities, a unit increase in the perceived importance of improvements to center-based services was associated with a 2.02 times greater likelihood of remaining in the community (odds ratio = 2.02, 95%CI [1.16, 3.53]). For caregivers, a unit increase in the perceived importance of improvements to financial subsidies was associated with a 2.80 times greater likelihood of remaining in the community (odds ratio = 2.80, 95%CI [1.26, 6.23]). The research hypotheses were partially supported, as only center-based services and financial subsidies contributed to self-efficacy for continuous community living among individuals with disabilities and caregivers, respectively.

## 4. Discussion

This study explored the connections between types of community support services and self-efficacy for continuous community living among groups of stakeholders. The findings could inform governmental initiatives for the promotion of community living with additional empirical support and insights for planning community support resources for specific groups. The current study also systematically compared and contrasted the levels of perceived importance of improvements to various community support services between care-recipients with disabilities and caregivers. As expected, one size did not fit all, given that the type of community support service might not necessarily yield the same benefits across groups of stakeholders. The specific needs of individuals with disabilities and caregivers should be taken into account when planning community support services for continuous community living.

The primary aim of the current study was to identify whether and how community support services contribute to self-efficacy for continuous community living among individuals with disabilities and caregivers. The analyzed support services included center-based, home-based, respite care, caregiver assistance, and financial subsidy services. In general, respondents were confident in their ability to continue living in the community, given adequate support services. Specifically, caregivers reported higher levels of the perceived importance of improvements to support services than individuals with disabilities. In this study, financial subsidies were found to be the most important resource to improve, regardless of the stakeholder. Moreover, financial subsidies were perceived by caregivers to be the most important service to improve for self-efficacy for continuous community living. This was not the case for individuals with disabilities, who perceived that center-based services were the most important to improve for self-efficacy for continuous community living. These effects remained unchanged after adjusting for gender, age, job status, and the presence of caregiving support.

The current study found that the majority of stakeholders reported self-efficacy for living in the community, provided that they had access to sufficient support services. To a certain extent, individuals with disabilities and caregivers typically embrace community living, but they may require the help of community support services and their quality of life could be enhanced by improvements to those support services [31]. Indeed, community living could be enhanced by comprehensive community support schemes, including mental health treatments, residential care, and financial subsidies [32]. Community living without the support of these services may lead to poorer outcomes for stakeholders due to unmet healthcare needs [8] or the even failure of community integration.

Other novel findings in our study were the differences in the perceived importance of improvements to community support services between the groups. Caregivers generally perceived a greater importance of improvements to support services than individuals with disabilities. Caregivers may have greater levels of awareness of the needs of care-recipients. As caregivers meet the needs of care-recipients within their own capacities, they have unique perspectives of caregiving processes. Moreover, caregivers may be more sensitive to the needs of care-recipients within the context of community living due to their proximity. Caregivers tend to look for appropriate resources that are available in the community to substitute for institutionalized care, support caregiving, and minimize caregiving burdens. The concerns of caregivers may also incorporate the concerns of care-recipients, reflected in their elevated awareness of the importance of improvements to the types of community support resources required for care-recipients to live in the community.

More importantly, both individuals with disabilities and caregivers reported that financial subsidies were the most important type of community support services that needed improvement. This finding suggested that both groups encountered financial difficulties that required increased financial support. To some extent, they could already be enduring economic hardships while living in the community, which could worsen in the long run, especially if carers gradually need to give up employment and rely on their savings [33,34]. Indeed, only about a third of the current sample were in employment and even they could need to rely on additional financial support to make ends meet. Consistent with prior studies, financial burdens were a major concern for community living [12,32]. Conversely, economic resources could build resilience and act as a buffer against stress related to disability [35,36,37]. Hence, policymakers should prioritize the financial needs of stakeholders over other types of community resources and implement relevant service voucher/insurance schemes similar to other countries, such as Australia [19].

Improvements to financial subsidies were rated by caregivers as the most important to maintain community living. Moreover, financial subsidies could also alleviate caregiver burdens, especially when transitioning to community living, for example, sponsorships to purchase assisted living equipment [38]. Moreover, caregivers are primarily concerned with the needs of care-recipients living in the community, who would otherwise likely be in institutionalized care. These observations need further corroboration in future studies. Future studies should also consider comparing and contrasting the impacts of direct financial support (i.e., cash) versus indirect financial sponsorship (e.g., vouchers) on general quality of life, as well as the integration of individuals with disabilities into the community.

With regard to self-efficacy for continuous community living, individuals with disabilities and caregivers had different priorities for improvements to community support services. The former prioritized improvements to center-based services, while the latter prioritized improvements to financial subsidies. Center-based services are multi-disciplinary and provide multiple functions that meet the various needs of care-recipients, for example, daily activities, educational programs, social events, mental health services, and vocational training [39,40,41]. Individuals with disabilities may use center-based services to expand their social network with peers, access professional help, and even develop skillsets that are necessary for community employment. These services are offered more frequently than typical home care services and provide greater service user satisfaction [42]. Conversely, other types of community support services may not offer such wide ranges of services compared to center-based services. In that sense, center-based services are more favorable for self-efficacy for continuous community living among individuals with disabilities.

### Limitations and Future Directions

Notwithstanding, the current study had several caveats. Firstly, the history of the living arrangements and service utilization of respondents was missing from the analyses. The stage of respondent deinstitutionalization or community living at the time of recruitment was not reported. Their past experiences or current satisfaction levels with community living and the actual service accessibility could influence their self-efficacy for community living. This additional data should be collected in future studies.

Secondly, the measures applied in the current study were not comprehensive. Self-efficacy for continuous community living was assessed using an item in the primary study, which only addressed a single aspect of community integration. Other facets of community integration were not included in the current study. Scholars should consider applying a validated scale to characterize the other domains involved in community integration to uncover clear associations between the types of services and community living. Additionally, due to space constraints, the current study focused primarily on quantitative data. The dynamics between community service utilization and community living processes remained largely unexplored. Future studies should consider using qualitative data to triangulate with the current set of findings.

Thirdly, the current study encountered a few challenges in terms of the data structures. The service factors and self-efficacy for continuous community living were confined to interpretation at the individual level, in that the individuals with disabilities and caregivers were treated as two independent groups in the analyses. The potential bias for data non-independence could not be corrected due to missing information. Scholars should consider recruiting dyads of care-recipients and caregivers in future studies to address the different concerns at a family level. Additionally, both caregivers and individuals with disabilities are often regarded as clinical samples with extended periods of exposure to distress, so data normality in their responses was hardly achieved.

Only individuals with disabilities and caregivers were included in the current study and it is not entirely clear whether and how other parties are involved in community integration. Given that community living typically involves other stakeholders, such as therapists, social workers, and case managers, and that these professionals have important roles throughout the community integration process, their input is indispensable to policymakers. Scholars should consider the experiences of multiple stakeholders in future studies.

## 5. Conclusions

The present investigation explored whether and how types of community support services contribute to self-efficacy for continuous community living among individuals with disabilities and caregivers. Respondents reported that their ability to remain in the community depended on access to sufficient support services. Improvements to center-based services were identified as being associated with increased self-efficacy for continuous community living among individuals with disabilities. Improvements to financial subsidies were identified as being associated with increased self-efficacy for continuous community living among caregivers. As the integration of stakeholders into the community hinges on resource allocation and disability care, the current findings could be particularly meaningful to policymakers. The findings suggest that public policies should prioritize center-based services for care-recipients and financial subsidies for caregivers to improve their self-efficacy for continuous community living. Finally, the types of financial subsidies that are the most effective at improving self-efficacy for community living among people with disabilities also need further investigation.

## Figures and Tables

**Table 1:** The characteristics of caregivers in our sample (*n* = 522).

Variables	*n*	%	*M*	*SD*
Sociodemographic Characteristics				
Age ^a^			4.76	1.22
Sex				
Female	413	79.1		
Male	109	20.9		
Has a full/part-time job				
No	358	68.6		
Yes	164	31.4		
Caregiving experience				
15 years or more of caregiving				
No	254	48.7		
Yes	268	51.3		
More than 12 h of caregiving a day				
No	311	59.6		
Yes	211	40.4		
Has several care-recipients with disabilities				
No	462	88.5		
Yes	60	11.5		
Is a voluntary caregiver				
No	332	63.6		
Yes	190	36.4		
Has a substitute caregiver if needed				
No	306	58.6		
Yes	216	41.4		
Characteristics of care-recipient(s)				
Has a care-recipient aged 20 or older				
No	322	61.7		
Yes	200	38.3		
Has care-recipient(s) without disabilities				
No	333	63.8		
Yes	189	36.2		
Perceived importance of improvement ^b,c^			4.43	0.03
Center-based services			4.42	0.57
Home-based services			4.35	0.76
Respite services			4.46	0.62
Caregiver assistance			4.41	0.62
Financial subsidies			4.55	0.61
Self-efficacy for continuous community living				
No	23	4.4		
Yes	499	95.6		

Notes: ^a^ 0 = younger than 10 years, 1 = 10–19 years, 2 = 20–29 years, 3 = 30–39 years, 4 = 40–49 years, 5 = 50–59 years, 6 = 60–69 years, 7 = 70–79 years, 8 = 80 years or above; ^b^ 1 = not important at all, 2 = slightly important, 3 = generally important, 4 = important, 5 = very important; ^c^ grand mean values and the standard deviation errors across types of services.

**Table 2:** The characteristics of individuals with disabilities in our sample (*n* = 948).

Variables	*n*	%	*M*	*SD*
Sociodemographic Characteristics				
Age ^a^			4.03	1.69
Sex				
Female	412	43.5		
Male	536	56.5		
Has a full/part-time job				
No	702	74.1		
Yes	246	25.9		
Has a voluntary caregiver				
No	493	52.0		
Yes	455	48.0		
Type of disability				
Attention deficit and hyperactivity disorders	33	3.5		
Autism spectrum disorders	124	13.1		
Hearing impairment	66	7.0		
Intellectual disabilities	250	26.4		
Mental illnesses	255	26.9		
Physical disabilities	250	26.4		
Special learning difficulties	21	2.2		
Speech impairment	57	6.0		
Vision impairment	78	8.2		
Visceral disabilities	26	2.7		
Perceived importance of improvement ^b,c^			4.14	0.02
Center-based services			4.09	0.74
Home-based services			4.10	0.98
Respite services			4.01	0.90
Caregiver assistance			4.09	0.77
Financial subsidies			4.35	0.77
Self-efficacy for continuous community living				
No	47	5.0		
Yes	901	95.0		

Notes: ^a^ 0 = younger than 10 years, 1 = 10–19 years, 2 = 20–29 years, 3 = 30–39 years, 4 = 40–49 years, 5 = 50–59 years, 6 = 60–69 years, 7 = 70–79 years, 8 = 80 years or above; ^b^ 1 = not important at all, 2 = slightly important, 3 = generally important, 4 = important, 5 = very important; ^c^ grand mean values and the standard deviation errors across types of services.

**Table 3:** Correlation matrices of caregivers (*n* = 522).

Variables	1	2	3	4	5	6	7	8	9	10	11	12	13	14	15
1. Age	-														
2. Sex	0.11 *	-													
3. Has a full/part-time job	−0.16 ***	0.20 ***	-												
4. 15 years or more of caregiving	0.45 ***	−0.06	−0.08	-											
5. More than 12 h of caregiving a day	−0.06	−0.20 ***	−0.26 ***	0.08	-										
6. Has several care-recipients with disabilities	−0.05	−0.01	−0.05	−0.01	−0.03	-									
7. Is a voluntary caregiver	0.00	0.08	0.11 **	0.00	−0.03	−0.01	-								
8. Has a substitute caregiver if needed	−0.07	0.09	0.11 *	−0.14 **	−0.16 ***	−0.02	0.08	-							
9. Has a care-recipient aged 20 or older	0.43 ***	0.11 *	0.04	0.09	−0.07	0.07	0.06	−0.05	-						
10. Has other care-recipient(s) without disabilities	−0.15 **	−0.09 *	−0.04	0.03	0.05	0.03	0.09 *	−0.04	−0.10 *	-					
11. Center-based services	−0.17 ***	−0.10 *	−0.04	0.03	0.03	−0.03	−0.01	−0.08	−0.24 ***	0.02	-				
12. Home-based services	−0.12 **	−0.03	0.06	0.02	0.03	−0.02	−0.01	−0.06	−0.06	0.03	0.55 ***	-			
13. Respite services	−0.01	−0.05	−0.03	0.17 ***	0.12 *	−0.08	−0.04	−0.18 ***	−0.15 **	−0.01	0.62 ***	0.64 ***	-		
14. Caregiver assistance	−0.22 ***	−0.11 **	−0.00	−0.05	0.03	0.03	−0.01	−0.07	−0.13 **	0.02	0.69 ***	0.43 ***	0.45 ***	-	
15. Financial subsidies	−0.17 ***	−0.05	−0.03	0.00	0.10 *	−0.04	−0.05	−0.11 **	−0.07	0.03	0.44 ***	0.46 ***	0.52 ***	0.51 ***	-
16. Self-efficacy for continuous community living	−0.14 **	−0.01	0.03	−0.10 *	0.04	0.02	0.01	0.01	−0.06	0.03	0.02	0.05	0.03	−0.00	0.12 *

* *p* < 0.05, ** *p* < 0.01, and *** *p* < 0.001.

**Table 4:** Correlation matrices of individuals with disabilities (*n* = 948).

Variables	1	2	3	4	5	6	7	8	9	10
1. Age	-									
2. Sex	−0.19 ***	-								
3. Has a full/part-time job	−0.24 ***	0.00	-							
4. Has a voluntary caregiver	−0.19 ***	0.11 **	0.03	-						
5. Center-based services	−0.06	−0.05	−0.02	−0.02	-					
6. Home-based services	0.03	−0.06	−0.05	0.01	0.61 ***	-				
7. Respite services	−0.02	−0.06	−0.06	0.01	0.69 ***	0.60 ***	-			
8. Caregiver assistance	−0.03	−0.07 *	0.00	0.00	0.68 ***	0.54 ***	0.58 ***	-		
9. Financial subsidies	0.03	−0.07 *	−0.02	−0.02	0.53 ***	0.48 ***	0.54 ***	0.58 **	-	
10. Self-efficacy for continuous community living	−0.01	0.05	−0.01	0.04	0.07 *	0.00	0.04	0.00	0.02	-

* p < 0.05, ** *p* < 0.01, and *** *p* < 0.001.

**Table 5:** Logistic regression models of self-efficacy for continuous community living (*N* = 1470).

	Caregivers (*n* = 522)				Individuals with Disabilities (*n* = 948)			
Variables	B	SE	OR ^a^	95% CI	B	SE	OR ^a^	95% CI
Sociodemographic Characteristics								
Age	−0.39	0.25	0.68	0.41–10.11	0.01	0.10	10.01	0.84–10.23
Male	0.09	0.57	10.10	0.36–30.32	0.38	0.31	10.46	0.79–20.70
Has a full/part-time job	0.16	0.55	10.18	0.40–30.49	−0.09	0.35	0.91	0.46–10.82
Caregiving experiences								
15 years or more of caregiving	−0.80	0.55	0.45	0.15–10.32	--	--	--	--
More than 12 h of caregiving a day	0.44	0.51	10.55	0.57–40.17	--	--	--	--
Has several care-recipients with disabilities	0.42	0.81	10.52	0.31–70.39	--	--	--	--
Is a voluntary caregiver	−0.01	0.48	0.99	0.39–20.53	0.40	0.32	10.50	0.80–20.79
Has a substitute caregiver if needed	0.03	0.47	10.03	0.41–20.60	--	--	--	--
Characteristics of (a) care-recipient(s)								
Has a care-recipient aged 20 or older	−0.15	0.52	0.86	0.31–20.40	--	--	--	--
Has other care-recipient(s) without disabilities	0.05	0.49	10.06	0.40–20.78	--	--	--	--
Perceived importance of improvement								
Center-based services	0.08	0.67	10.08	0.29–40.01	0.70 *	0.28	20.02	10.16–30.53
Home-based services	0.08	0.36	10.09	0.54–20.20	−0.23	0.20	0.79	0.54–10.16
Respite services	−0.10	0.50	0.91	0.34–20.43	0.10	0.24	10.10	0.69–10.76
Caregiver assistance	−0.86	0.54	0.43	0.15–10.23	−0.38	0.28	0.68	0.40–10.18
Financial subsidies	10.03 *	0.41	20.80	10.26–60.23	0.04	0.24	10.04	0.65–10.66

Notes: ^a^ odds ratios; * *p* < 0.05.

## Data Availability

Restrictions apply to the availability of these data. Data were obtained from the Labor and Welfare Bureau (LWB) of the Government of the Hong Kong Special Administrative Region of the People’s Republic of China (HKSAR, PRC) and are available from the authors with the permission of the LWB.

## Appendix A

	**Center-Based**	**Home-Based**	**Respite Services**	**Caregiver Assistance**	**Financial Subsidies**
1. To increase the number of District Support Centers (DSCs) for persons with disabilities	☑				
2. To increase the number of Parent/relative Resource Centers (PRCs)				☑	
3. To increase the number of Community Rehabilitation Day Centers (CRDCs)	☑				
4. To increase the number of Social and Recreational Centers for the Disabled (S&RCs)	☑				
5. To increase the number of Support Centers for Persons with Autism (SPA)	☑				
6. To increase the quota of Home Care Services		☑			
7. To increase the quota of Day Training Services	☑				
8. To increase the quota of Day Respite Services			☑		
9. To increase the quota of Residential Respite Services			☑		
10. To provide individuals with disabilities/caregivers with cash subsidies					☑
11. To sponsor individuals with disabilities and families to purchase assistive technology devices					☑
12. To provide caregivers with emotional support services and disability care skills training				☑	
